# Influence of glass-ceramics transmittance on chemomechanical properties of light- and dual-cure resin cements

**DOI:** 10.1590/0103-644020256670

**Published:** 2025-12-08

**Authors:** Roberta Cristina Costa Guimarães, Dayane de Oliveira, Mateus Garcia Rocha, Jean-François Roulet, Saulo Geraldeli, Mario Alexandre Coelho Sinhoreti

**Affiliations:** 1 Department of Restorative Dentistry, Piracicaba Dental School, University of Campinas, Piracicaba, SP, Brazil.; 2 Department of Restorative Dental Science, College of Dentistry, University of Florida, Gainesville, FL, USA.; 3 Division of Biomedical Materials, School of Dental Medicine, East Carolina University, Greenville, NC, USA.

**Keywords:** glass-ceramics, resin cements, light attenuation, physicochemical properties

## Abstract

This study assessed the effects of light attenuation by glass-ceramics (IPS e.max Press, IPS e.max CAD, IPS Empress CAD, and Vita Suprinity) on the degree of conversion (DC), polymerization shrinkage stress (PSS), and microshear bond strength (µSBS) of dual-cure (RelyX Universal and RelyX Ultimate) and light-cure (Variolink N LC and Variolink Esthetic LC) resin cements. Ten slabs (12 × 12 × 1.5 mm) were fabricated from each ceramic. For light attenuation analysis (n=1), the ceramic slab was positioned onto the light-curing tip (Valo-Cordless), and the radiant emittance (mW/cm²) was measured. Resin cement cylinders (n=10) were fabricated using Tygon tubes positioned onto ceramic slabs, water-stored for 24 h, and tested for µSBS. For the PSS test, glass rods were positioned 1 mm apart, and the gap was filled with resin cement, which was light-cured. The rod displacement was considered for the calculation of PSS. The DC test (n=3) for each resin cement was conducted using a spectrometer. Surface morphology was examined by SEM (LEO 435 VP, 20 kV) at 5.000×. DC, PSS, and µSBS data were analyzed using ANOVA and Tukey’s test (α = 0.05). No significant difference was observed among the resin cements for PSS. Variolink Esthetic LC showed a µSBS mean value statistically lower than RelyX Ultimate. For Suprinity, the light-cure cements showed DC mean values significantly lower than the dual-cure cements. It was possible to conclude that the ceramic slabs significantly increased the light attenuation, which affected the DC, PSS, and µSBS of all the dual- and light-cure resin cements.

## Resumo

Este estudo avaliou os efeitos da atenuação da luz por vitrocerâmicas (IPS e.max Press, IPS e.max CAD, IPS Empress CAD e Vita Suprinity) sobre o grau de conversão (GC), Tensão de contração de polimerização (TCP) e resistência de união por microcisalhamento (µRUC) de cimentos resinosos de duais (RelyX Universal, RelyX Ultimate) e fotopolimerizáveis (Variolink N LC, Variolink Esthetic LC). Dez placas (12 × 12 × 1,5 mm) foram confeccionadas de cada cerâmica. Para a análise da atenuação da luz (n = 1), a placa cerâmica foi posicionada sobre a ponteira do aparelho fotopolimerizador (Valo-Cordless), e a irradiância (mW/cm²) foi medida. Cilindros de cimento resinoso (n = 10) foram confeccionados utilizando tubos de Tygon posicionados sobre a dentina, fotoativados através das placas cerâmicas, armazenados em água por 24 h e testados quanto à µRUC. Para o teste de TCP, hastes de vidro foram posicionadas com 1 mm de distância entre elas, e o espaço foi preenchido com cimento resinoso, que foi fotopolimerizado. O deslocamento das hastes foi considerado para o cálculo da TCP. O teste de GC (n = 3) para cada cimento resinoso foi realizado com um espectrômetro de infravermelho por transformada de Fourier. Os dados de GC, TCP e µRUC foram analisados por ANOVA e teste de Tukey (α = 0,05). Não foram observadas diferenças significativas entre os cimentos resinosos para o TCP. O Variolink Esthetic LC apresentou valor médio de µRUC estatisticamente inferior ao RelyX Ultimate. Para a Suprinity, os cimentos fotopolimerizáveis apresentaram valores médios de GC significativamente inferiores aos cimentos de dupla cura. Concluiu-se que as cerâmicas aumentaram significativamente a atenuação da luz, o que afetou o GC, o TCP e a µRUC de todos os cimentos resinosos fotopolimerizáveis e duais.

## Introduction

Ceramic, involving different brands and chemical compositions, is widely used as a restorative material in dentistry, mainly due to its natural appearance, effectively mimicking natural teeth concerning color and light interaction^1^. The success of indirect glass-ceramic restorations relies on the type of ceramic, its bond strength to the resin cement and tooth, and its light transmittance to the resin cement^1^ ^,^ ^2^. Clinical studies have reported satisfactory long-term outcomes or leucite-reinforced, lithium disilicate, and fluorapatite glass-ceramics^2^ ^,^ ^3^ ^,^ ^4^. Despite the clinical longevity of many glass-ceramics, long-term performance depends on how effectively the resin cements cure beneath the restoration. This, in turn, is determined by how much light reaches the resin cement layer and which part of the spectrum (violet vs. blue) is available to activate the photoinitiator^1^ ^,^ ^4^. Insufficient curing compromises the integrity of the adhesive interface and is associated with marginal discoloration, postoperative sensitivity, debonding margins, and failure of the indirect restoration, especially as ceramic thickness and opacity increase and when the available spectrum is less effective for the photoinitiator used^5^.

Glass-ceramics with a leucite content of approximately 50 vol.% were developed to improve their mechanical properties, especially for use in single-unit crowns^1^. Their physicochemical properties rely on the size and volume (%) of crystals in their glassy matrix, the finer the crystals, the stronger the material^1^. The translucence of leucite-based glass-ceramics, available in pressable and machinable (CAD/CAM) versions, depends on their chemical composition (percentage of crystals).

A glass-ceramic containing lithium disilicate has been developed. The size and volume of its crystals (lithium disilicate) have been adjusted to provide better mechanical and physical properties^1^. This ceramic is translucent due to the relatively low refractive index of the lithium disilicate crystals. The machinable version is provided in a partially crystallized block, a condition that facilitates the machining process^6^. Some glass-ceramics have been innovated by changing the crystal structure of lithium silicate and adding zirconium oxide to its chemical composition^1^.

The difference in thickness, chemical composition, and color of glass-ceramics might impact the light transmittance during the polymerization of the resin cement and thus affect its mechanical and clinical performance^5^. Light cure units (LCUs) emit different wavelengths to initiate the polymerization process^7^. Some resin materials require LCUs with wavelengths in the violet range (380~420 nm) to activate alternative photoinitiators^8^. Insufficient radiant emittance from the LCU might reduce the degree of conversion of the resin cement and its bond strength to the dental substrate, especially in light-cure resin cements^9^.

The present study evaluated the effect of four glass-ceramics (1.5-mm thick) with different compositions on the physicochemical properties (degree of conversion, polymerization shrinkage stress, and bond strength to dentin) of light- and dual-cure resin cements. The null hypotheses were: (i) the different ceramics would have no impact on the radiant emittance and, consequently, on the degree of conversion, the polymerization shrinkage stress, and the bond strength of the resin cements; and (ii) the curing modes (dual- and light-cure) of the resin cements would have no impact on their degree of conversion, polymerization shrinkage stress, and bond strength.

## Materials and methods

### Study Design

**Figure 1:**
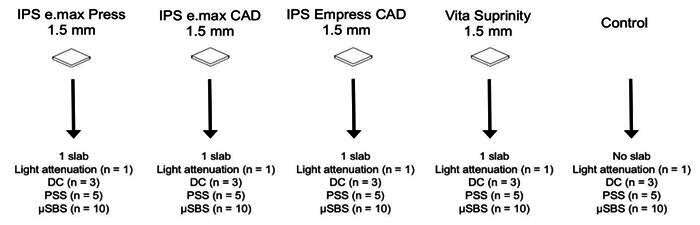
A glass-ceramic slab was used to fabricate the resin cement specimens.

This study included two light-cure, Variolink N LC (Ivoclar Vivadent, Schaan, Liechtenstein) and Variolink Esthetic LC (Ivoclar Vivadent), and two dual-cure resin cements, RelyX Universal (3M Oral Care, St. Paul, MN, USA) and RelyX Ultimate (3M Oral Care). Table 1 shows technical information about the resin cements. Glass-ceramic (IPS e.max Press, IPS e.max CAD, IPS Empress CAD, and Vita Suprinity - shade HT A2) slabs (1.5 mm in thickness; n = 10) were fabricated and tested for light attenuation, micro-shear bond strength, degree of conversion, and polymerization shrinkage stress. The glass-ceramic slabs were assigned to three groups according to the type of ceramic and resin cement (Figure 1). The control involved no ceramic slab during light curing. Power analysis was conducted to determine the sample size for each test (power = 0.8 or above) at a significant level of 0.5 (β = 0.2).

**Table 1:** Shade, type, composition, and manufacturers of the resin cements.

Material	Shade	Type	Composition*	Manufacturer
Variolink N LC	T	Light cure	Barium glass, ytterbiumtrifluoride, BisGMA, UDMA, TEGDMA, Si-Zr mixed oxide, barium glass filler, camphorquinone as photoinitiator, tertiary amine, and pigments.	Ivoclar, Vivadent, Schaan, Liechtenstein
Variolink Esthetic LC	Neutral	Light cure	Bis-GMA, UDMA, TEGDMA, ytterbium trifluoride, boroaluminofluorosilicate glass, spheroidal mixed oxide, Ivocerin as photoinitiator, and pigments.	Ivoclar, Vivadent, Schaan, Liechtenstein
RelyX Universal	A1	Dual cure	BPA derivative-free dimethacrylate and phosphorylated dimethacrylate adhesion monomers, photoinitiator system, novel amphiphilic redox initiator system, radiopaque fillers, rheological additives, and pigments	3M Oral Care, St Paul, MN, USA
RelyX Ultimate	A1	Dual cure	Base Paste: Methacrylate monomers, radiopaque silanated fillers, Initiator components, stabilizers, and rheological additives; Catalyst Paste: Methacrylate monomers, radiopaque alkaline (basic) fillers, initiator components, stabilizers, pigments, rheological additives, and fluorescence dye dark cure	3M Oral Care, St Paul, MN, USA

* Information provided by the manufacturers.

### Glass-ceramic slabs

Lithium disilicate glass-ceramic (IPS e.max Press; shade A2, HT) was used to fabricate one slab (12 x 12 mm and 1.5 mm thick) from a model of Duralay acrylic resin (Reliance Dental, Alsip, IL, USA). The resin model was positioned into a silicone cylinder filled with phosphate-bonded universal investment (IPS PressVest premium, Ivoclar Vivadent, Liechtenstein). After the investment hardened, the silicone cylinder was removed, and the investment cylinder was placed into a furnace (Vulcan A-550, Degussa-Ney, Yucaipa, CA, USA) at 850 °C for one hour to burn out the acrylic resin. The investment cylinder, along with the glass-ceramic ingot, was placed into another furnace (Programat EP 3010, Ivoclar Vivadent) at 915 °C for 15 min to inject the ceramic into the investment mold. After cooling, the ceramic slab was removed from the investment and sandblasted with 100-μm aluminum oxide (Special Jet Medium, Ivoclar Vivadent) at 2-bar pressure. The slab had one side polished (#600 and #1200 sandpaper) under running water for 60 s and was submitted to an ultrasound bath (GT Sonic, Shenzhen, Guangdong, China).

One slab (1.6 mm thick) was obtained from each other CAD ceramic material - IPS e.max CAD ceramic block (shade A2, HT; Ivoclar Vivadent, Schaan, Liechtenstein), IPS Empress CAD ceramic block (shade A2, HT; Ivoclar Vivadent, Liechtenstein), and Vita Suprinity ceramic block (shade A2, HT; Vita Zahnfabrik, H. Rauter GmbH & Co., Bad Säckingen, Germany) - using a low-speed diamond disc (Isomet 1000 Precision Saw; Buehler Co., Lake Bluff, IL, USA) under water cooling and sintered in a furnace (Austromat 647; Dekema, Freilassing, Germany) according to the manufacturer’s instructions. The slabs had one surface sandpapered (#600 and #1200) under running water for 60 s to obtain a thickness of 1.5 mm, as checked with a digital caliper (Mitutoyo, Tokyo, Japan), and were then submitted to an ultrasound bath for 15 min (GT Sonic, China).

### Light attenuation analysis

The radiant emittance (mW/cm²) of the curing light unit (Valo cordless, Ultradent Products, South Jordan, UT, USA) was calculated by dividing the radiant power (mW) - measured with a power meter (Ophir Optronics, Jerusalem, Israel) - by the area of the light tip (0.63585 cm²; 0.9 cm in diameter). The spectral distribution data were obtained using a spectrometer (USB2000, Ocean Optics, Dunedin, FL, USA). Light attenuation was calculated considering the ceramic slab (1.5 mm thick) positioned onto the curing light tip compared to the control (no ceramic slab).

### Degree of conversion analysis

The degree of conversion (DC) of the resin cements (n = 3) was measured using an ATR-FTIR spectrometer (Nicolet iS20, Thermofisher, Waltham, MA, USA). The unpolymerized resin cements were placed within a Teflon mold (ø = 4 mm, 0.2 mm thick) placed on the diamond ATR detector of the FTIR spectrometer, scanned, and then light cured (Valo cordless, Ultradent Products) for 20 s through the respective ceramic slab and without the slab (control). The polymerized resin cements were scanned again 300 seconds after light curing. The unconverted carbon double bonds (C=C) were quantified by calculating the ratio derived from the vinyl aliphatic (1638 cm^−1^) to the aromatic absorption area (1608 cm^−1^) signals for both polymerized and unpolymerized specimens. The DC of the resin cements was calculated according to the following equation:

DC % = 1- Xa/Ya  Xb/Yb× 100

Where Xa (polymerized) and Xb (unpolymerized) represent the bands of the polymerizable aliphatic double bonds, and Ya (polymerized) and Yb (unpolymerized) represent the bands of the aromatic double bonds.

### Polymerization shrinkage stress

The polymerization shrinkage stress (PSS) of the resin cements (n = 5) was analyzed through photographic images obtained with the aid of a universal testing machine (Instron 4411, Instron, Canton, MA, USA)^10^. Glass rods (13- and 54-mm long and 4 mm in diameter) had their surfaces sandpapered (#600 grit), etched with 10% hydrofluoric acid for 20 s, and coated with Monobond N silane (Ivoclar Vivadent). The 54-mm glass rod was held in the upper fixture attached to the load cell of the universal testing machine (Instron 4411, Instron, USA), while the 13-mm glass rod was held in the lower fixture. The testing equipment consists of a slot that allows the placement of the light-curing unit tip in contact with the 13-mm glass rod. The upper and lower glass rods were aligned 1 mm apart. The camera was positioned perpendicularly to the space (1 mm) between the glass rods to calculate their displacement (µm), considering three images taken in different periods - before and after the resin cement placement, and after the photoactivation. The compliance of the testing equipment was calculated (1.66 µm/N; C-factor of 0.5) to adjust the nominal stress values^11^ ^,^ ^12^.

Each resin cement was placed in the space (1 mm thick) between the glass rods and light-cured (Valo cordless, Ultradent Products) through the ceramic slab and then without the slab (control). The photoactivation time was increased to 25 s to compensate for the 20% radiant emittance attenuation as the curing light passed through the lower rod^10^. The rod displacement (µm) recorded 10 min after light curing was used to calculate the PSS of each resin cement. The displacement was divided by the maximum nominal strength (N) recorded on the universal testing machine at compliance of 0.4 μm/N^13^. A previously reported formula^13^ was used to calculate the maximum polymerization shrinkage stress (MPa) by summing the PS^nominal^ and PS^corrected^ and dividing the maximal nominal strength by the cross-sectional area of the glass rod.

*Nominal Polymerization Stress PSnominal = Forceuniversal testing machine (N)*

*Corrected Polymerization Stress PScorrected = Strain µm X ComplianceapparatusCompliancecavity*

### Microshear bond strength (µSBS)

Two hundred bovine incisors had their buccal surface enamel abraded (SiC sandpaper: #300, #400, and #600 grit; Buehler, Lake Bluff, IL, USA) to expose the dentin. After enamel abrasion, the roots of the incisors were removed using a diamond disc (Isomet 1000 Precision Saw, Buehler Co., Lake Bluff, IL, USA) under water cooling. The teeth (leaving the dentin uncovered) were then embedded in acrylic resin (ProBase Cold, Ivoclar Vivadent, Liechtenstein) within polyvinyl chloride tubes and cleaned in an ultrasonic bath (Thermo Fisher Scientific, Waltham, MA, USA) for 10 min.

The embedded dentin specimens were assigned to twenty groups based on the type of ceramic (IPS e.max Press, IPS e.max CAD, IPS Empress CAD, Vita Suprinity) and the resin cement (RelyX Ultimate, RelyX Universal, Variolink Esthetic LC, and Variolink N LC), including the control (n = 10). For all groups, the dentin surface was coated with a universal adhesive (Single Bond Universal Plus - 3M) according to the manufacturer's instructions and then light-cured for 10 s.

**Figure 2:**
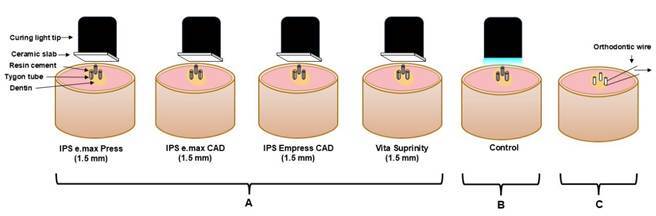
(A) Light curing through the ceramic slabs, (B) no slab (control), and (C) the cement cylinders bonded to the dentin and looped with an orthodontic wire for the µSBS test.

Three Tygon tubes (0.75 mm in diameter x 0.3 mm long) were placed onto the dentin surface of each specimen, filled with the resin cements tested using an exploratory probe and light cured (Valo cordless, Ultradent Products) through the ceramic slab (1.5 mm thick) for 20 s (Figure 2A), considering the control (no slab, Figure 2B). The Tygon tubes containing the bovine teeth were then stored in an incubator immersed in distilled water for 24 h at 37 °C. The tubes were then removed using a scalpel blade (Swann-Morton, Sheffield, England), and the cement cylinders had their adhesive interface checked for defects using an optical stereomicroscope (Meiji Techno Co., Ltd., Japan).

The three cement cylinders bonded to the dentin were placed in a µSBS testing device mounted on a universal testing machine (Instron 4411, Instron, Canton, MA, USA). A 0.2-mm-in-diameter stainless steel orthodontic wire was looped around each cylinder and aligned with the adhesive interface (Figure 2C). The µSBS test was then carried out at a 1-mm/min crosshead speed until failure. The mean value of the three cylinders was calculated for statistical analysis. A stereomicroscope (Meiji Techno Co., Ltd., Japan) was used to analyze the dentin and classify the failure modes as (a) adhesive, (b) cohesive in dentin, (c) cohesive in resin cement, and (d) mixed (two or three failures combined).

### SEM analysis of ceramic microstructure

To evaluate the surface morphology of the ceramic microstructures, one specimen from each group was etched with 10% hydrofluoric acid for 20 seconds, rinsed under running water for 1 minute, and subsequently cleaned in an ultrasonic bath for 10 minutes. After cleaning, the specimens were mounted on metallic stubs and coated with a gold layer using a sputter coater (Balzers-SCD 050, Balzers Union, Liechtenstein) at a current of 40 mA for 180 seconds. Surface analysis was performed by a single operator using a scanning electron microscope (SEM; LEO 435 VP, Cambridge, UK) operating at 20 kV. Images were acquired at a magnification of 5,000×.

### Statistical analysis

The data concerning all tests were submitted to the Shapiro-Wilk test to check for normality and to Levene’s test to verify the homoscedasticity of variances. The µSBS and PSS data were analyzed by two-way ANOVA and Tukey's post-hoc test (α = 0.05). The two factors analyzed were the ceramic slab (IPS e.max press, IPS e.max CAD, IPS Empress CAD, Vita Suprinity, and control) and the resin cements (Variolink N LC, Variolink Esthetic LC, RelyX Universal, and RelyX Ultimate). DC data were submitted to one-way ANOVA and Tukey's post-hoc test (α = 0.5), considering the ceramic slab (IPS e.max press, IPS e.max CAD, IPS Empress CAD, Vita Suprinity, and control) as a factor. The emission spectra (mWcm^2^/nm) data and the failure modes were submitted to a non-statistical descriptive analysis.

## Results

### Light attenuation analysis

**Figure 3:**
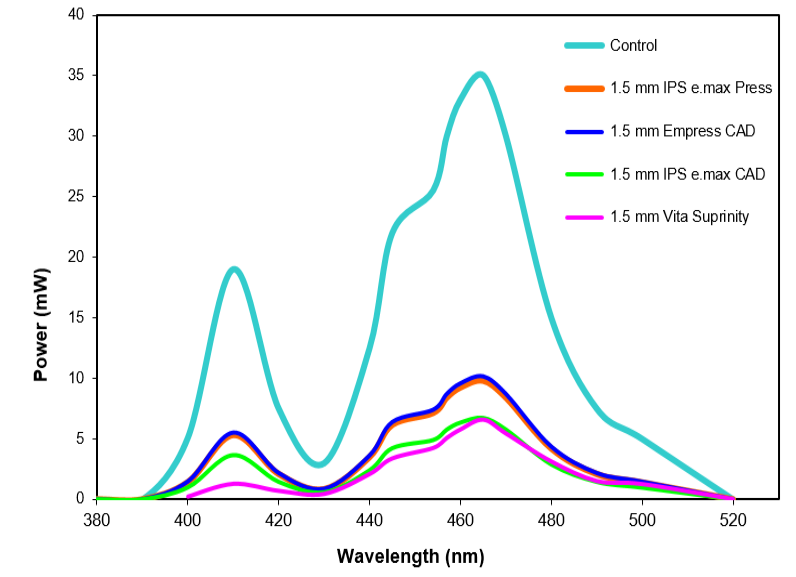
Light attenuation caused by the ceramic slabs, considering the spectral radiant semittance (mW/cm^2^/nm) of the multiwave LED cure unit (Valo cordless).

The LED curing light wavelength ranges were 380-420 nm for the violet and 420-520 nm for the blue light (Table 2 and Figure 3). The spectrophotometer analysis, considering the blue and violet spectra, showed a reduction in radiant emittance for all the ceramic slabs. In the control (no ceramic slab), a radiant emittance of 1060 mW/cm² (blue: 879 mW/cm²; violet: 181 mW/cm²) was recorded. The ceramic slabs caused a reduction in the radiant emittance - 366 mW/cm² for the IPS e.max Press, 299 mW/cm² for the IPS e.max CAD, 371 mW/cm² for the IPS Empress CAD, and 285 mW/cm² for the Suprinity (Table 2). Table 2 also shows the percentage (in relation to control) of radiant emittance transmitted through each ceramic slab, considering the blue and violet spectra. Figure 3 illustrates the light attenuation caused by the ceramic slabs according to the spectral radiant emittance (mW/cm^2^/nm).

**Table 2:** Radiant emittance values (mW/cm^2^) through each ceramic slab.

Wavelength	Control (no ceramic slab)	IPS e.max Press	IPS e.max CAD	IPS Empress CAD	Suprinity
Violet 380-420 nm	181	37 (20.4%)	27 (14.9%)	38 (21.0%)	20 (11.1%)
Blue 420-520 nm	879	329 (37.4%)	272 (30.9%)	333 (37.8%)	265 (30.1%)
Total (380-520 nm)	1060	366 (34.5%)	299 (28.2%)	371 (35.0%)	285 (26.8%)

(%) Percentage of radiant emittance in relation to the control.

### Degree of conversion (DC) analysis

Table 3 shows the DC mean values (%) and standard deviations obtained for each resin cement, considering the different types of ceramic and the control (p = 0.001). The control (no slab) concerning all resin cements showed DC mean values significantly higher than those obtained for the ceramic slab groups. No significant difference in DC was observed among the slab groups concerning RelyX Ultimate and RelyX Universal. Variolink N LC and Variolink Esthetic LC light-cured through the IPS e.max Press and IPS Empress CAD slabs showed DC mean values significantly higher than those obtained for the Suprinity. IPS e.max CAD showed no significant difference in DC among the slab groups.

**Table 3:** DC mean values (%) and standard deviation for the resin cements, considering the ceramics and control.

	RelyX Ultimate	RelyX Universal	Variolink N LC	Variolink Esthetic LC
Control	58.10 ± 0.92 a	65.05 ± 1.02 a	55.77 ± 0.89 a	56.97 ± 1.67 a
IPS e.max Press	55.06 ± 0.88 b	58.66 ± 1.13 b	52.92 ± 0.95 b	51.31 ± 1.07 b
IPS e.max CAD	55.16 ± 0.51 b	57.93 ± 1.09 b	52.22 ± 1.06 bc	50.56 ± 0.84 bc
IPS Empress CAD	54.60 ± 1.09 b	59.17 ± 1.33 b	52.68 ± 0.70 b	51.89 ± 1.07 b
Suprinity	54.55 ± 1.55 b	57.62 ± 1.04 b	51.21 ± 0.82 c	48.56 ± 1.23 c

Different lowercase letters indicate statistically significant differences in columns (p < 0.05).

### Polymerization Shrinkage Stress (PSS)

Table 4 shows the PSS mean values (MPa) and standard deviations for all the resin cements, considering the different ceramic slab types and the control (p = 0.005). In the control group (no slab), RelyX Universal and Variolink Esthetic LC showed PSS mean values that were significantly higher than those observed for RelyX Ultimate and Variolink N LC. Concerning the groups with the slab, no significant difference in PSS mean values was observed among the resin cements and ceramics. RelyX Universal, Variolink N LC, and Variolink Esthetic LC (control: no slab) showed PSS mean values significantly higher than those obtained for the slab groups. RelyX Ultimate showed no significant difference in PSS among the slab groups and the control.

**Table 4:** PSS mean values (MPa) and standard deviation for the resin cements light cured through ceramic slabs and control.

	RelyX Ultimate	RelyX Universal	Variolink N LC	Variolink Esthetic LC
Control	5.56 ± 0.51 a, B	7.67 ± 0.77 a, A	5.69 ± 0.24 a, B	7.41 ± 0.67 a, A
IPS e.max Press	4.34 ± 0.62 a, A	4.91 ± 0.66 b, A	3.67 ± 0.36 b, A	3.86 ± 0.25 b, A
IPS e.max CAD	5.02 ± 1.49 a, A	5.16 ± 0.82 b, A	4.34 ± 1.50 b, A	4.86 ± 1.92 b, A
IPS Empress CAD	5.12 ± 1.28 a, A	5.35 ± 1.48 b, A	4.63 ± 0.67 b, A	5.06 ± 0.79 b, A
Suprinity	4.88 ± 0.16 a, A	5.03 ± 0.26 b, A	4.01 ± 0.66 b, A	4.27 ± 0.32 b, A

Different lowercase letters indicate statistically significant differences in columns (p < 0.05).

Different uppercase letters indicate statistically significant differences in rows (p < 0.05).

### Microshear bond strength (µSBS)

Table 5 shows the µSBS mean values and standard deviation for the resin cements tested, considering the glass-ceramic slabs and control (no slab). No significant difference (2-way ANOVA: p = 0.068) in µSBS was observed in the interaction of the two factors (ceramics and cements). Considering the pooling means values, the control (no ceramic slab) showed µSBS mean values significantly higher than those observed for the ceramic groups (Tukey’s test: p = 0.001). A significant difference was observed between RelyX Ultimate and Variolink Esthetic LC (Tukey’s test: p = 0.013). RelyX Ultimate showed the highest µSBS mean values (pooling mean value) among the resin cements, while Variolink Esthetic LC showed the lowest.

**Table 5:** µSBS mean values (MPa) and standard deviation for the resin cements, considering the glass-ceramics (slabs).

Glass-ceramics	Resin cements				
	RelyX Universal	RelyX Ultimate	Variolink N LC	Variolink Esthetic LC	Pooling mean
Control (no slab)	22.69 ± 3.22	20.79 ± 1.60	23.18 ± 3.77	20.62 ± 2.39	21.82 ± 2.98 a
IPS e.max Press	14.26 ± 3.55	17.67 ± 5.54	13.54 ± 2.84	12.27 ± 2.71	14.44 ± 4.19 b
IPS e.max CAD	13.72 ± 3.00	16.05 ± 3.04	12.18 ± 3.30	11.80 ± 2.87	13.44 ± 3.38 b
IPS Empress CAD	14.27 ± 1.77	16.46 ± 3.17	12.97 ± 4.11	12.34 ± 1.86	14.01 ± 3.20 b
Vita Suprinity	14.66 ± 4.02	15.67 ± 3.07	13.20 ± 4.03	12.16 ± 2.49	14.01 ± 3.20 b
Pooling mean	15.92 ± 4.70 AB	17.32 ± 3.45 A	15.01 ± 5.66 AB	13.83 ± 4.42 B	-

Mean values followed by different lowercase letters in the column and capital letters in the row indicate statistical differences (Tukey's test).

**Figure 4:**
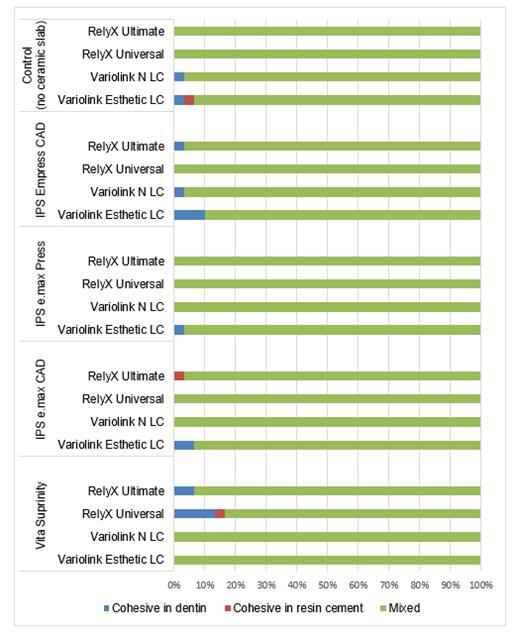
Rate (%) of failure modes for each resin cement, considering each glass-ceramic slab and control (no slab).

Figure 4 shows the rate (%) of failure modes for each resin cement, considering the ceramics (slabs) and control (no slab). Mixed failures were predominant in all groups, including the control group.

**Figure 5:**
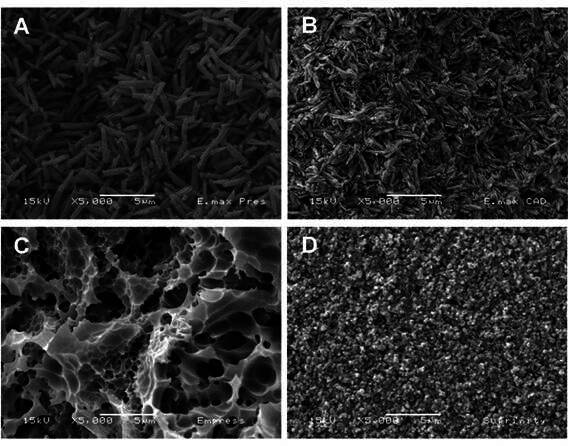
SEM images after etching with 10% hydrofluoric acid: A - E.max Press: Lithium disilicate crystals are exposed following the removal of the glassy matrix by acid etching. B - E.max CAD: A greater number of lithium disilicate crystals are exposed, and they appear smaller than those in E.max Press. C - Empress: Extensive dissolution of the vitreous phase leads to the extrusion of leucite crystals as they detach from the matrix. D - Vita Suprinity: The surface shows irregularities, with numerous microporosities and grooves caused by the dissolution of the glassy phase.

Figure 5 shows SEM images of the ceramic specimens after etching with 10% hydrofluoric acid. In Figure 5A, the surface of the E.max Press ceramic-primarily composed of lithium disilicate is shown following etching. The etching process has selectively removed the glassy matrix surrounding the crystalline phase, effectively exposing the elongated lithium disilicate crystals. These crystals are distinguishable and exhibit a relatively larger size. Figure 5B illustrates the etched surface of E.max CAD, revealing a greater density of lithium disilicate crystals than E.max Press. However, these crystals appear significantly smaller and more numerous, suggesting that the microstructure of E.max CAD contains finer crystallites dispersed within the glass matrix. Figure 5C shows the Empress ceramic surface, demonstrating pronounced dissolution of the vitreous phase after etching, resulting in the apparent extrusion and isolation of leucite crystals. These crystals seem to be detaching or may have already detached from the surrounding matrix. Vita Suprinity (Figure 5D) reveals distinct irregularities characterized by visible microporosities and grooves. These features are attributed to the dissolution of the glassy phase surrounding the zirconia-reinforced lithium silicate structure.

## Discussion

All the ceramics tested caused an expressive reduction in the radiant emittance, especially for the violet light (Table 2 and Figure 3). This finding is in accord with those reported in previous studies investigating light transmittance attenuation through ceramics^14^ ^,^ ^15^ ^,^ ^16^ ^,^ ^17^. Plus, it might account for the significant reduction in DC (Table 3), PSS (Table 4), and bond strength (Table 5) observed for the resin cements in the ceramic slab groups. Based on these findings, the first null hypothesis was rejected.

The light source used in this study was a polywave light-curing unit (VALO-Cordless) whose emission covers the violet (≈380-420 nm) and blue (≈420-495 nm) ranges, with distinct peaks that activate violet-sensitive photoinitiators (e.g., Ivocerin/TPO) and camphorquinone (blue-sensitive)^18^. In polywave units, irradiance across the tip is not uniform; combined with the greater attenuation and scattering of shorter wavelengths (violet) by ceramics, this tends to deliver more blue light to the resin cement^15^. This source/ceramic effect explains results, attenuation of violet produced lower DC and µSBS for resin cements relying on violet-sensitive initiators, whereas the blue light was less affected.

Light attenuation has been reported to be associated with light reflection, absorption, and diffusion, all of which might be directly related to the blue and violet spectra of the curing light - the shorter (violet) wavelength, the greater the light attenuation^18^ ^,^ ^19^. This phenomenon can be explained by Rayleigh scattering, where the intensity of scattered light is inversely proportional to the fourth power of the wavelength (λ⁻⁴). Consequently, shorter wavelengths, such as violet light, experience pronounced scattering when interacting with nanometric pores and microstructural features within ceramics, leading to increased light attenuation and reduced penetration depth^19^. In the present study, when compared with the violet spectrum (380-420 nm), the blue light spectrum (420-495 nm) resulted in less attenuation (Table 2), making it more effective in penetrating the ceramics, especially for Suprinity. This reduction in light transmittance has been reported to compromise the polymerization process of resin cements and their bond strength over time, affecting their clinical performance^2^ ^,^ ^4^ ^,^ ^20^.

Light attenuation and spectrum ranges are also associated with the chemical composition of ceramics. IPS Empress CAD and IPS e.max Press showed lower light attenuation than that observed for IPS e.max CAD and Suprinity (Table 2). This finding might be due to the different crystalline structures (filler and glass matrix) of the ceramics^21^. IPS e.max Press and IPS e.max CAD comprise 70% of randomly oriented, interlocking plate-like lithium disilicate crystals; light transmittance through lithium disilicate ceramics is reduced by the plate-like crystals^21^ ^,^ ^22^. The crystals in IPS e.max CAD are smaller than those in IPS e.max Press [Lithium Disilicate: The Future of All-Ceramic Dentistry], a condition that might interfere with light transmittance (Figure 5A and B). IPS Empress CAD contains approximately 50 vol.% of crystals and glass matrix^21^ ^,^ ^22^, a condition that might account for its lower light attenuation. Suprinity is a lithium-silicate-based ceramic to which zirconium oxide was added to improve its mechanical properties^23^; zirconium oxide might have been responsible for its greater light attenuation (Figure 5D), especially in the violet wavelength.

The light attenuation reduced the polymerization process and might account for the reduction in DC, PSS, and µSBS mean values obtained for all the resin cements (Tables 3 and 5) when compared with the control, a finding in accord with those previously reported^14^ ^,^ ^24^ ^,2)^. In this study, light-cured resin cements Variolink N LC and Variolink Esthetic LC include a photoinitiator in their formula, which makes these cements light-dependent. Variolink N LC has camphorquinone as a photoinitiator sensitive to blue light at 470 nm peak, and Variolink Esthetic LC has an alternative photoinitiator (Ivocerin), which is sensitive to violet light^26^ ^,^ ^27^. With the reduction in the light transmittance caused by the ceramics, especially with Suprinity, a significant decrease in DC mean values was observed with these light-cured resin cements. Despite the reduction in DC mean values ​​for both light-cured cements, only the Variolink Esthetic LC cement showed a reduction in µSBS values. This material includes in its formula an alternative photoinitiator (Ivocerin), which is sensitive to violet light^26^ ^,^ ^27^, which was significantly attenuated when Suprinity ceramic was used. The peculiar nature of violet light has a direct impact on the degree of conversion of cementation materials that react to this light spectrum due to its lower penetration, resulting in a reduced degree of conversion and consequently affecting the bond strength between the resin cement and the ceramic, as elucidated in the present study and previous studies^5^ ^,^ ^14^ ^,^ ^18^ ^,^ ^25^ ^,^ ^26^.

For the dual-cured resin cements (RelyX Ultimate and RelyX Universal), Suprinity did not cause a reduction in DC mean values compared to the other ceramics. Dual-cured resin cements have demonstrated the ability to compensate for the light exposure reduction induced by ceramics, ensuring effective polymerization even when ceramics reduce the light intensity^18^ ^,^ ^25^. This occurs due to the dual-cure mechanism, in which polymerization is initiated both by light activation and by a chemical reaction between the peroxide and amine components of the two-paste system^28^. The chemical polymerization is initiated by a redox reaction between benzoyl peroxide and a tertiary amine. Upon mixing the two components, benzoyl peroxide decomposes to form benzoyloxyl radicals, which react with the tertiary amine to produce reactive free radicals. These free radicals initiate the polymerization of methacrylate monomers, resulting in the formation of a crosslinked polymer network that ensures the proper curing and mechanical stability of the resin cement, even in the absence of light exposure^18^ ^,^ ^20^ ^,^ ^24^ ^,^ ^28^.

The data presented in Table 4 indicate that the different ceramic slabs reduced the PSS values in the tested resin cements. RelyX Universal and Variolink Esthetic LC in the control (no ceramic slab) recorded the highest PSS mean values. This finding may be due to the low molecular weight of the new (BPA-free) monomers in RelyX Universal and its low filler particle ratio (20-35%), according to its manufacturer (Safety Data Sheet, 3M™ RelyX™ Universal). The high PSS mean value obtained for Variolink Esthetic LC when light-cured without the ceramic slab (control) may be due to its photoinitiator Ivocerin, which is highly reactive and has a high molar extinction coefficient^5^ ^,^ ^26^ ^,^ ^29^. Due to its efficient absorption of light energy and rapid initiation of polymerization, Ivocerin promotes accelerated monomer conversion^30^. This process increases polymerization shrinkage stress^31^. However, when 1.5-mm ceramic slabs were used, there was a significant reduction in PSS mean values. This finding may be related to the reduction in radiant emittance and DC mean values for all resin cements.

Despite the advances in cementation materials over recent years, the disadvantages related to PSS in resin cements remain a recurring issue^18^ ^,^ ^28^. PSS in indirect restorations can result in postoperative sensitivity and marginal displacement and contribute to the appearance of marginal staining. It is worth noting that these stains are occasionally used as criteria for replacing indirect restorations^28^. Therefore, the second null hypothesis, which suggested that dual-cure and light-cure resin cements would show no difference in the degree of conversion, polymerization shrinkage stress, and bond strength, was rejected.

The absence of a statistically significant difference in bond strength and polymerization shrinkage stress among the different types of ceramics used at the same thickness (1.5 mm) can be attributed to several factors. Although there was a difference in the microstructure of the IPS e.max Press, IPS e.max CAD, IPS Empress CAD, and Suprinity ceramics, the uniformity of the ceramic thickness (1.5 mm) in both groups reduced its influence on light attenuation, which explains the absence of a statistically significant difference between them. Mixed failures are considered more favorable, as they suggest a more uniform stress distribution along the adhesive interface^18^. In contrast, a predominance of adhesive failures is indicative of a weaker bond, which may be attributed to inadequate polymerization of the resin cement or surface treatment^18^. These findings are consistent with previous studies indicating that as ceramic thickness increases, light transmission is attenuated, which can compromise the resin cement polymerization and increase the prevalence of adhesive failures^18^. Evaluating both bond strength and failure modes is important, as it provides an accurate assessment of the adhesive performance of resin cements in ceramic restorations. These combined factors suggest that, when used at equal thicknesses, the types of ceramics tested in this study do not exhibit notable discrepancies in terms of bond strength, polymerization shrinkage stress, and DC (only for the dual-cure resin cements). However, it is important to emphasize that conclusions may vary depending on the material thickness^32^.

After 10% HF etching, SEM images revealed distinct microstructural features across all ceramics (Figure 5A-D). IPS e.max Press and IPS e.max CAD showed densely packed, elongated lithium disilicate crystals within a glassy matrix, with e.max CAD having finer and more numerous crystals than e.Max Press. IPS Empress CAD displayed a glass-rich matrix with dispersed leucite crystals, with selective dissolution of the vitreous phase partially exposing leucite after HF etching. Vita Suprinity (zirconia-reinforced lithium silicate) exhibited a fine, highly crystalline microstructure with microporosities caused by the dissolution of the glassy phase around lithium silicate crystals containing ZrO₂. These crystalline phases scatter light, attenuating shorter wavelengths (violet), which helps explain the greater loss of radiant emittance, especially for Suprinity, and the related reductions in DC and µSBS when violet-sensitive photoinitiators are used (Figure 3; Tables 2, 3, 4-5).

One limitation of this in vitro study is that the ceramic thickness used (1.5 mm) and the different types of ceramics tested do not fully reflect the challenges encountered during adhesive cementation in vivo, such as surface treatment, occlusion, and moisture control. Light attenuation and the subsequent effects on resin cements can vary significantly depending on the ceramic material and shade selected^32^. Another point to consider is that the study evaluated a limited number of resin cements, which may limit the generalization of the results to all cements available on the market. Therefore, future studies should include a broader range of resin cements to provide a more comprehensive understanding of their performance under different conditions. This study did not assess microhardness, color stability, or long-term mechanical (e.g., fatigue/aging) properties that may also be influenced by light attenuation through ceramics. Future studies should address these outcomes under different ceramic thicknesses and shades to better delineate their clinical impact.

The results of the present study suggest that light-cured resin cements may be suitable for thinner and more translucent ceramic restorations, where light transmission can be achieved to ensure adequate polymerization. However, as ceramic thickness increases or when using opaque materials, dual-cured resin cements become an option to compensate for the reduction in light transmittance and ensure polymerization^16^. This observation is in accordance with manufacturer recommendations that suggest the use of dual-cured cements for restorations above 2 mm in thickness or for opaque ceramics. Moreover, conducting clinical trials to validate the in vitro findings and provide more clinically relevant data to guide material selection and clinical procedures is essential. From a clinical perspective, ceramic-dependent light attenuation should guide adhesive cementation strategies. For thin and/or translucent indirect restorations, light-cure resin cements predominantly activated by violet light (e.g., Ivocerin-based) may provide adequate curing. When the ceramic is thicker or opaquer and primarily attenuates violet wavelengths, less light reaches the resin cement; therefore, to guarantee complete curing, it is recommended to use dual-cure resin cements, prioritize blue-sensitive photoinitiators, properly position the curing tip, and, if necessary, extend the curing time. In practice, this helps reduce the risk of undercuring, marginal discoloration, and decreased bond strength^9^ ^,^ ^31^.

## Conclusion

The ceramic slabs significantly increased the light attenuation, affecting the conversion degree, polymerization shrinkage stress, and microshear bond strength of all the dual- and light-cure resin cements tested. The effect of the light attenuation was less intense on the mechanical properties of RelyX Ultimate.
